# Temporal muscle thickness is not a prognostic predictor in patients with high-grade glioma, an experience at two centers in China

**DOI:** 10.1515/med-2024-1053

**Published:** 2024-10-30

**Authors:** Yunlong Pei, Haixiao Jiang, Enpeng Zhang, Boming Xia, Lun Dong, Yan Dai

**Affiliations:** Department of Critical Care Medicine, The Affiliated Hospital of Yangzhou University, Yangzhou, China; Department of Neurosurgery, The Affiliated Hospital of Yangzhou University, Yangzhou, China; Department of Neurosurgery, Dalian Medical University, Dalian, China; Department of Emergency, Sir Run Run Shaw Hospital, Zhejiang University, Hangzhou, China; Department of Neurosurgery, Northern Jiangsu People’s Hospital, No. 98 Nantong, Westroad, 225001, Yangzhou, Jiangsu, China; Medical Research Center, Northern Jiangsu People’s Hospital, No. 98 Nantong Westroad, 225001, Yangzhou, Jiangsu, China

**Keywords:** temporal muscle thickness, glioblastoma, overall survival

## Abstract

Temporal muscle thickness (TMT) serves as an indicator of sarcopenia and holds predictive value for various cancers. This study aims to evaluate the prognostic significance of TMT for high-grade glioma patients. A retrospective review of 172 high-grade glioma patients from January 2015 to December 2022 was conducted. TMT value was measured based on contrast-enhanced T1-weighted magnetic resonance images before surgery. Pearson analysis was used to evaluate potential correlations. Cox regression analysis was performed to evaluate overall survival for high-grade glioma patients. In our study, the cutoff value of TMT was determined as 7.4 mm. TMT value was not a significant prognostic predictor for high-grade glioma patients (hazard ratio [HR]: 1.151, 95% confidence interval [CI]: 0.9299–1.424, *p* = 0.196). World Health Organization (WHO) VI and high body mass index (BMI) value were significantly associated with poorer survival outcomes (HR: 2.6689, 95% CI: 1.5729–4.528, *p* < 0.001; HR: 1.120, 95% CI: 1.0356–1.211, *p* = 0.005). TMT did not show a significant association with other factors (*p* > 0.05). Notably, age demonstrated a significant difference between the thicker and thinner groups (*p* = 0.019). Our study revealed that WHO grade and BMI demonstrated significant prognostic value for survival outcomes. Consequently, TMT does not appear to be a significant or applicable predictor in patients with high WHO grades.

## 1 Introduction

Glioblastoma (GBM), one of the most prevalent and malignant tumors, has a poor prognostic outcome with a short median survival of about 14.6 months and a 5-year survival rate of less than 10% [1,2,3]. Despite the implementation of advanced treatment modalities, including maximal safe surgical resection, radiation therapy, and temozolomide drug treatment [4,5,6], the median survival of GBM patients remains short [7]. Consequently, there is an escalating need to assess the prognosis of GBM patients. Several risk factors were identified by previous studies, such as age, World Health Organization (WHO) grade, radiologic findings, surgery type, molecular characteristics, and postoperative chemoradiotherapy [8,9,10,11].

Originally introduced by Baumgartner to evaluate the age-associated reductions in muscle mass among older adults, sarcopenia, determined as a performance of reduced muscle strength and mass, was widely acknowledged as a significant risk factor in oncology providing a prognostic value to assess overall survival (OS) for patients with pancreatic cancer, liver cancer, renal carcinoma, and colorectal cancer [12,13,14,15,16].

Temporal muscle thickness (TMT), a novel radiographic feature of sarcopenia, has been employed for evaluating patient outcomes [17,18,19,20]. Previous studies, such as the work by Ranganathan et al. [17], have shown that mean TMT was significantly correlated with age (*r* = −0.36, *P* < 0.001) and total psoas muscle area (*r* = 0.57, *P* < 0.001). However, the prognostic utility of TMT for GBM patients remains a subject of controversy [21]. While several studies suggested that patients with a high level of TMT were frequently associated with longer OS [20,22,23,24], others argued that there was not a significant relationship between TMT and OS in GBM patients [25,26]. Furthermore, unreliable conclusions may draw due to the bias in study design, patient selection, and follow-up time.

Therefore, this study aimed to evaluate the prognostic significance of TMT for high-grade glioma patients.

## 2 Methods

### 2.1 Patients

Clinical data for glioma patients were retrospectively collected from Subei Hospital and The Affiliated Hospital of Yangzhou University between January 2015 to December 2022. The inclusion criteria comprised: (1) a pathological diagnosis of glioma cancer and (2) the absence of other malignant tumors. Patients with insufficient clinical and follow-up data were excluded. Finally, this study included 172 glioma patients.

### 2.2 Data collection

The clinical data were recorded as follows: WHO grade, gender, age, body mass index (BMI), smoking, alcohol consumption, tumor diameter, fibrinogen (FIB), albumin-to-globulin ratio (AGR), gama-glutamyltransferase (GGT), monocyte-to-lymphocyte ratio (MLR), neutrophil-to-lymphocyte ratio (NLR), and platelet-to-lymphocyte ratio (PLR). Ethical approval and consent were obtained for this study.

**Figure 1:**
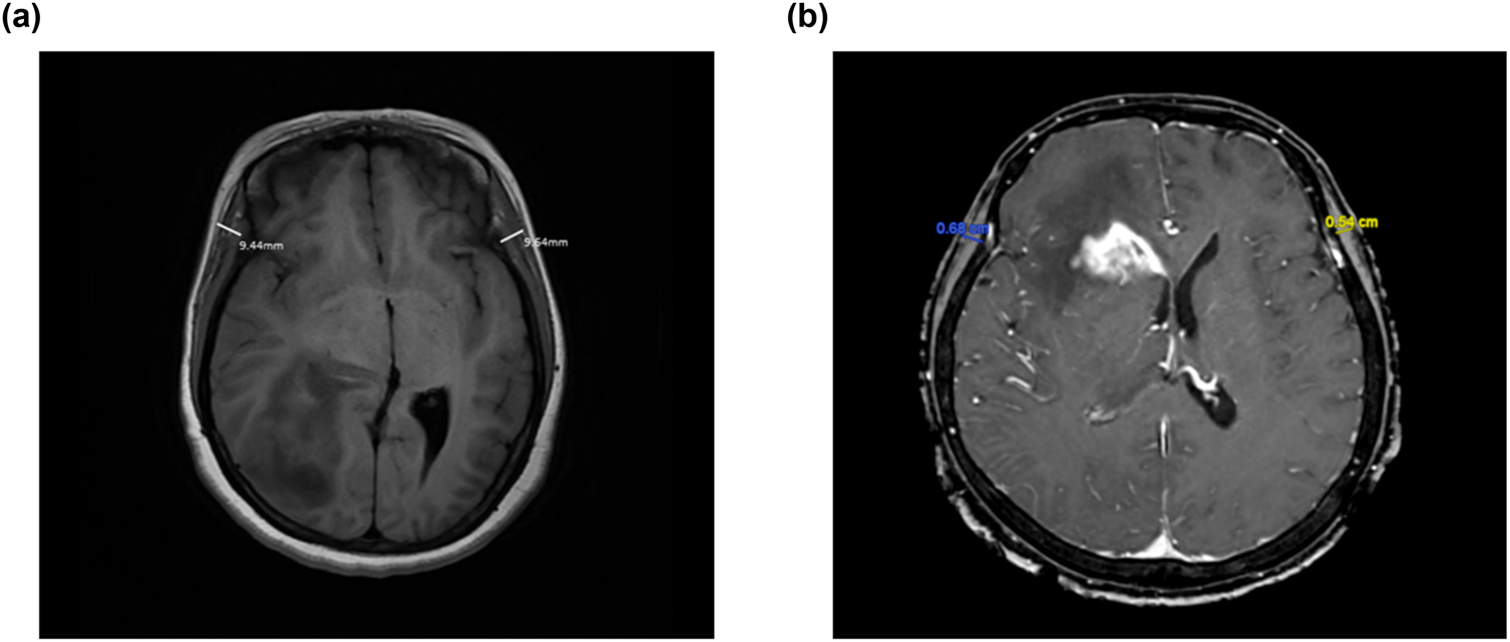
The measurement of TMT value. (a) A 56-year-old male patient with an OS of 30.5 months (TMT = 9.54 mm). (b) A 50-year-old male patient with an OS of 26 months (TMT = 6.10 mm).

The measurement of TMT was evaluated by preoperative T1-weighted MR images with 1 mm axial thin slices (1.5 or 3.0 scanners). The plane was selected parallel to the anterior commissure-posterior commissure line and perpendicular to the long axis of the temporal muscle. TMT levels were measured on both sides, based on the orbital roof and the Sylvian fissure, and the mean value was the final result. Two examples are shown in Figure 1.

### 2.3 Follow-up

Post-operative follow-up for all glioma patients was conducted every 3 months during the first 2 years, and subsequently every 6 months thereafter. For patients who did not have a scheduled hospital review, telephone communication was utilized to collect follow-up data. The endpoint, OS, was defined as the duration from the date of surgery to either the date of death or the date of the last follow-up.

### 2.4 Statistical analysis

R software (Version 3.6.3; https://www.R-project.org) and X-tile [27] were used for statistical analysis. X-tile was used to identify the cutoff value of TMT. Pearson analysis was used to evaluate potential correlations. The univariate and multivariable Cox regression analyses were carried out to identify significant prognostic predictor. *P* < 0.05 is considered to be statistically significant.


**Ethical approval:** The study followed the Declaration of Helsinki’s ethical guidelines. Additionally, this study was supported by the Medical Ethics of The Affiliated Hospital of Yangzhou University (2023-YKL03-G042) and Subei Hospital (2021ky138-1).
**Informed consent:** All patients signed informed consent.

## 3 Results

### 3.1 Basic characteristics of included patients

A total of 172 high-grade patients were included in this study from January 2015 to December 2022. The basic characteristics of included patients are given in Table 1. There were 138 patients diagnosed with WHO VI and 34 glioma patients were WHO III. The glioma patients consisted of 94 males, and the median age of patients was 55.5 years. The median BMI was 27.77 and the median OS was 15.2 months for glioma patients.

**Table 1:** Basic characteristics of included patients

	Thinner cohort ≤7.4 mm	Thicker cohort >7.4 mm	*p* value
WHO			
III	19	15	0.689
VI	72	66	
Gender			
Male	50	44	0.935
Female	41	37	
**Age**	52.35 ± 14.36	56.99 ± 10.89	**0.019**
BMI	24.51 ± 2.79	25.07 ± 2.61	0.129
Smoking			
Yes	66	54	0.403
No	25	27	
Alcohol consumption			0.343
Yes	19	21	
No	72	60	
Diameter	5.3 ± 1.82	4.98 ± 1.70	0.754
FIB	3.06 ± 0.94	2.98 ± 0.79	0.156
Albumin	43.41 ± 4.85	42.48 ± 5.48	0.343
Globulin	24.95 ± 4.48	24.66 ± 4.59	0.827
AGR	1.79 ± 0.37	1.78 ± 0.33	0.698
GGT	25.28 ± 17.37	27.74 ± 18.78	0.399
MLR	0.34 ± 0.29	0.3 ± 0.22	0.081
NLR	4.93 ± 4.69	4.08 ± 3.32	0.054
PLR	153 ± 97.32	142.33 ± 65.93	0.144

FIB, fibrinogen; AGR, albumin-to-globulin ratio; GGT, gama-glutamyltransferase; MLR, monocyte-to-lymphocyte ratio; NLR, neutrophil-to-lymphocyte ratio; PLR, platelet-to-lymphocyte ratio.

### 3.2 TMT analysis

No significant correlation was observed between TMT values and various clinical indicators. The results of Pearson correlation analysis indicated that mean TMT was not significantly associated with gender, age, BMI, tumor diameter, FIB, albumin, globulin, AGR, GGT, MLR, NLR, and PLR (*p* > 0.05).

**Figure 2:**
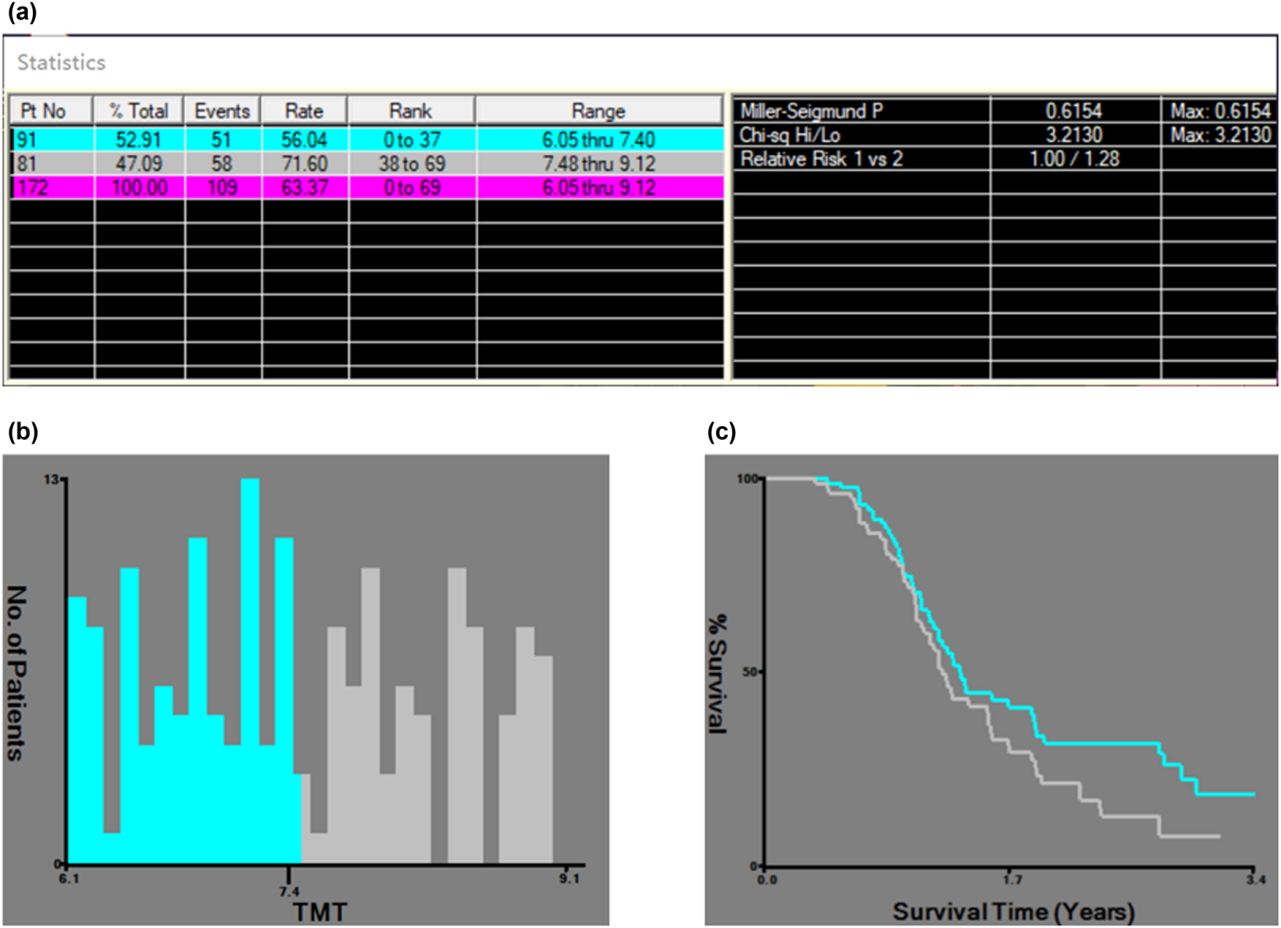
The TMT cutoff value was determined by X-tile. (a) The optimal cutoff for the variable TMT was determined to be 7.4 mm (*p* = 0.6154). (b) Histogram showing the distribution of patients in the two groups. (c) Kaplan-Meier survival curves for the two patient groups divided by the cutoff point, illustrating a divergence in survival rates over time.

X-tile was employed to determine the TMT cutoff value, suggesting a cutoff value of 7.4 mm for mean TMT (Figure 2). Subsequently, glioma patients were categorized into the thinner cohort (TMT ≤ 7.4 mm) and the thicker cohort (TMT > 7.4 mm) (Table 1). The results indicated that only age is significantly different between the thinner cohort and thicker cohort (*p* = 0.019). The analysis of the included clinical indicators between the two groups revealed no significant differences for other predictors.

**Figure A1:**
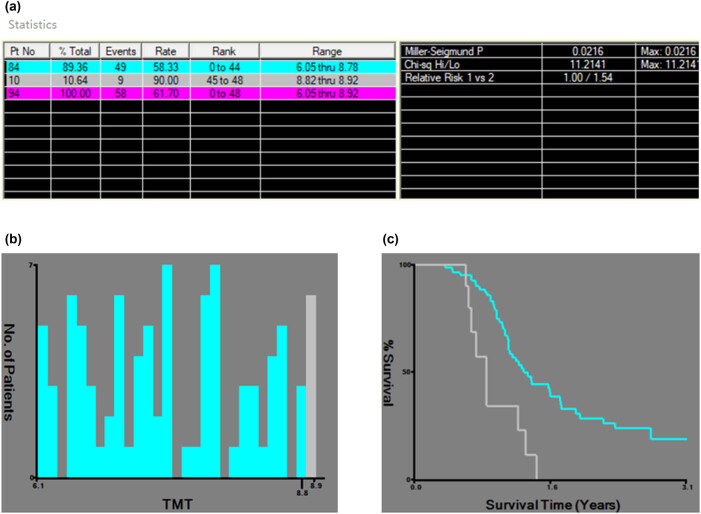
The male TMT cutoff was 8.8 mm.

**Figure A2:**
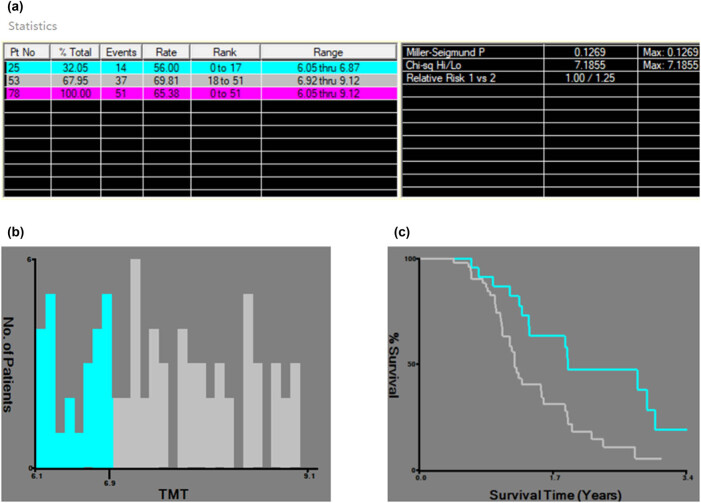
The female TMT cutoff value was 6.9 mm.

We also evaluate the male and female TMT cutoff values by X-tile (Figures A1 and A2). The results suggested that a male TMT cutoff value was 8.8 mm, and a female TMT cutoff value was 6.9 mm.

### 3.3 Cox regression analysis

**Figure 3:**
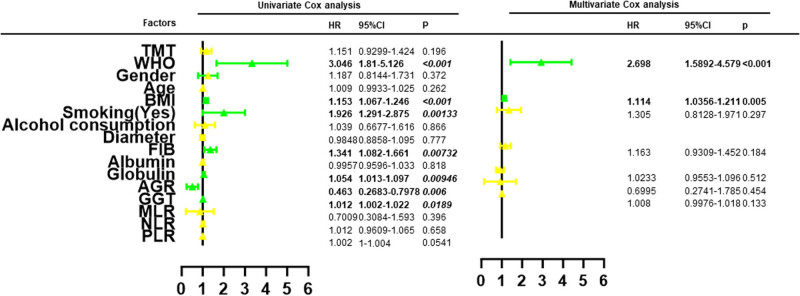
The results of univariate and multivariate Cox analysis.

Univariate and multivariable Cox regression analyses were conducted to assess the predictive significance of clinical risk factors (Figure 3). The results indicated that TMT was not a significant clinical predictor for high-grade glioma (hazard ratio [HR]; 1.151, 95% confidence interval [CI]; 0.9299–1.424, *p* = 0.196). Finally, WHO grade and BMI values were identified by Cox regression analysis (HR; 2.698, 95% CI; 1.5892–4.579, *p* < 0.001; HR; 1.114, 95% CI; 1.0295–1.206, *p* = 0.007). The cut-off point for BMI was determined to be 27.7, as calculated by X-tile. Glioma patients with WHO VI and higher BMI were significantly associated with poorer survival outcomes.

### 3.4 Subgroup analysis

We did a subgroup analysis to evaluate TMT predictive value for different survival outcomes (Table 2). Glioma patients were divided into survival more than 2 years and less than 2 years, as only five glioma patients survived more than 5 years. The results indicated that TMT did not present a significant predictive ability (*p* > 0.05). We also performed Cox regression analysis using data from glioma patients with more than median survival years and less than median OS. The results indicated that TMT was not a significant risk factor for glioma patients (*p* > 0.05).

**Table 2:** Subgroup analysis of TMT value for glioma patients

	Univariate Cox regression		
	HR	95% CI	*p* value
**Survival year**			
≥2 years	1.333	0.6087–2.918	0.473
<2year	0.994	0.8025–1.231	0.957
**Median OS**			
>15.2 month	1.290	0.7748–2.148	0.327
<15.2 month	1.147	0.9021–1.457	0.264

OS, overall survival.

## 4 Discussion

Sarcopenia has recently been identified as a significant biomarker for evaluating survival outcomes in various diseases [28,29,30,31,32]. Typically, the skeletal mass index, measured at the third lumbar vertebra muscle according to CT findings, is one of the most common methods for evaluating sarcopenia [33,34]. The skeletal muscle mass could be performed to predict prognosis for various cancers using the results of radiological findings, such as colorectal cancers, gastric cancers, hepatocellular carcinoma, and pancreatic cancers [28,29,30,31,35,36,37,38]. A previous study reported that the cross-sectional area of lumbar skeletal muscle and TMT level were significantly prognostic risk factors in lung cancer and melanoma patients with brain metastases [35]. However, the lumbar muscle measurement of the cross-sectional area takes a relatively long time. Additionally, patients with craniocerebral tumors do not take routine abdominal CT or MRI examination, and additional scanning will increase the economic burden and radiation dose of patients. Therefore, novel biomarkers should be researched and developed to evaluate sarcopenia for brain cancers.

Recently, TMT has been applied to predict the prognostic performance of cancer patients. As a novel non-invasive biomarker for sarcopenia, TMT was significantly correlated with OS for patients accompanied with non-small-cell lung cancer, melanoma, and breast cancer brain metastases [35,39,40]. Furtner et al. [39] found that compared with melanoma patients with a TMT level below the cut-off point, those with a TMT level above the cut-off point demonstrated better prognostic ability, with survival times of 13 vs 5 months (*p* < 0.001). One study reported that TMT was a significant independent prognostic predictor for brain metastasis patients from breast cancer (HR: 0.791, 95% CI; 0.703–0.889, *p* < 0.001) and lung cancer (HR: 0.710, 95% CI; 0.646–0.780, *p* < 0.001) [40]. Ilic and his colleagues [41] demonstrated that a combination of TMT and modified frailty index showed a significant predictive value for patients with lung cancer and surgically treated metastasis by assessing sarcopenia and preoperative frailty. Patients with brain metastases often present a long disease history and cause related complications such as muscle wasting. In addition, the long-term disease could influence metabolism response, which might reduce the skeletal muss mass index.

However, the role of TMT in evaluating the clinical outcome of glioma patients remains controversial. Our study reveals negative results regarding TMT’s predictive ability, stemming from two institutional experiences. Within our two-center cohort, TMT was not a significant prognostic risk factor (HR: 1.151, 95% CI; 0.9299–1.424, *p* = 0.196). Consistent with our findings, several studies have reported that TMT level was not significantly associated with OS for glioma patients. Muglia and his colleagues [25] suggested that TMT level did not present a substantial association with age and performance status, and the HR value for OS was 1.34 (95% CI; 0.68–2.63, *p* = 0.403). Klingenschmid et al. [26] demonstrated that TMT did not significantly predict functional outcomes in patients with high-grade glioma. Clinical Frailty Scale and Karnofsky Performance Scale exhibited superior prognostic value compared to TMT. Only one factor, female gender, is significantly associated with TMT. In our study, the results of Pearson correlation analysis revealed no significant correlation between TMT and any factors. Furthermore, there was no statistically significant difference observed between the thinner group and the thicker group for the included factor, except for age (*p* = 0.019).

According to the results observed, it leads us to speculate that TMT might not exhibit a robust prognostic prediction ability for patients with high-grade glioma. One potential explanation could be the frequent occurrence of developing neurological symptoms in high-grade patients. The overall thickness of body muscles may undergo changes; yet, TMT may not show significant alterations when glioma patients undergo cranial MRI scans. Moreover, abdominal CT scans are typically reserved for only a subset of glioma patients. Additionally, the relationship between thickness of TMT and lumbar vertebra muscle, serving as a skeletal mass index for evaluating sarcopenia, remains unclear. Both parameters may decrease in high-grade glioma patients, but the extent of this decrease requires further investigation. As a result, the prognostic value of TMT remains questionable. Traditional functional scores, WHO classification, and pathological factors appear to hold more promise in providing beneficial prognostic insights.

Nevertheless, our study has several limitations that warrant acknowledgment. First, the major limitations are the relatively small sample size, particularly as the study was conducted across two centers. Further investigation through multi-center studies is imperative to enhance the generalizability of our findings. Second, there are significant prognostic risk factors among glioma patients, such as other radiologic features, isocitrate dehydrogenase mutation status, 1p/19 codeletion, and immune cell function.

## 5 Conclusion

In this study, TMT did not demonstrate a significant association with other factors in high-grade glioma patients. Compared with BMI and WHO classification, TMT does not appear to have a significant impact on predicting prognosis for glioma patients.
